# Targeted transarterial embolization of the femoral head: development of a minimally invasive approach to model Legg-Calvé-Perthes disease in piglets

**DOI:** 10.1371/journal.pone.0323360

**Published:** 2025-05-14

**Authors:** Susan A. Novotny, Jennifer C. Laine, Casey P. Johnson, Alexandra R. Armstrong, Erick O. Buko, Ashton A. Amann, Reza Talaie, Ferenc Tóth

**Affiliations:** 1 Gillette Children’s Specialty Healthcare, Saint Paul, Minnesota, United States of America; 2 Department of Family Medicine and Community Health, University of Minnesota, United States of America; 3 Department of Orthopedic Surgery, University of Minnesota, United States of America; 4 Department of Veterinary Clinical Sciences, University of Minnesota, United States of America; 5 Center for Magnetic Resonance Research, University of Minnesota, United States of America; 6 Vascular and Interventional Radiology, Department of Radiology, University of Minnesota, United States of America; University of Life Sciences in Lublin, POLAND

## Abstract

Clinical management of children with Legg-Calvé-Perthes Disease (LCPD) is hampered by incomplete understanding of how the extent of ischemic injury and the duration and quality of subsequent repair determine patient outcome. The traditional piglet model of LCPD is limited to capturing global femoral head ischemia; thus, a new model is needed in which the extent of ischemia can be varied to replicate the spectrum of disease seen in children. In this exploratory study, we used an iterative approach to test and refine methods to bilaterally occlude vessels supplying the femoral heads in n = 8 young piglets under angiographic control. The *deep* and/or *acetabular medial femoral circumflex arteries* (DMFCA and AMFCA) were identified and embolized using either embolic particles or liquid embolic agents. The extent of ischemia was assessed immediately post-embolization (4 piglets) and/or 7 days following embolization (7 piglets) using contrast-enhanced magnetic resonance imaging (CE-MRI). After the final CE-MRI, piglets were euthanized, and their femora were harvested for histologic evaluation. Embolization of the DMFCA alone caused transient ischemia that largely resolved by 7 days with small regions of fibrovascular repair of ischemic injury remaining on histology. Embolization of both the DMFCA and AMFCA resulted in a greater degree of pathologic changes at 7 days post-operatively, but also with nearly complete restoration of femoral head perfusion. We found that combining injection of embolic particles with subsequent placement of an embolic micro-coil was the most effective approach to induce ischemic injury, which may be aided in larger piglets. While our findings should be interpreted cautiously due to the wide range in the age and size of animals investigated, they demonstrate that transarterial embolization of the vascular supply of the femoral head results in transient ischemia and histological changes consistent with partial ischemic injury. These results will inform further development of a minimally invasive piglet model of LCPD that offers a unique representation of the spectrum of pathophysiology of LCPD compared to the traditional model.

## Introduction

Legg-Calvé-Perthes disease (LCPD) is a pediatric hip disorder caused by idiopathic disruption of the blood supply to the growing femoral head that carries the risk of early onset osteoarthritis and lifelong disability [1]. Clinical management of children with LCPD is hampered by limited understanding of how the extent of ischemic injury, the duration and degree of revascularization and bone resorption, and the quality of reossification determine patient outcome. In the absence of this critical information, a wide variety of treatments are often prescribed to manage patients with LCPD. Children younger than 6 years old are usually treated non-operatively, and treatments may include periods of non-weightbearing, bracing, physical therapy and casting. Conversely, treatment of children older than 6 years of age frequently involves surgery [1]. Unfortunately, these interventions often fail to prevent the development of femoral head deformity, as demonstrated by two large prospective studies showing that 49% [2] and 51% [3] of children with LCPD develop an ovoid or flat femoral head, which are known to progress to moderate to severe OA in 61% of patients [4]. Improving clinical outcomes in LCPD patients is challenging due to the heterogeneity and relatively low incidence of the disease, the years-long course of healing, and limited access to pediatric tissue samples. Advancing patient care therefore requires an animal model that can replicate the wide range of severity of LCPD to allow development of novel diagnostic and treatment approaches.

The traditional LCPD piglet model uses an open surgical approach to the hip joint combined with transection of the ligamentum teres and its artery, to allow hip subluxation and placement of an encircling ligature around the femoral neck to interrupt the vascular supply [5]. While this animal model has been instrumental in improving our understanding of the pathogenesis and treatment of LCPD [5–18], there are multiple aspects of this model that poorly represent LCPD as it is seen in children. First, hip arthrotomy and subluxation are invasive and may induce an altered healing response in the model, unlike the spontaneous onset of disease in children. Second, the importance of the artery of the ligamentum teres (foveal artery) in the pathogenesis and subsequent healing of LCPD has been recognized [19,20], thus its transection in the traditional LCPD model may interfere with accurately capturing the disease. Lastly, the current model is limited to induction of global femoral head ischemia, without the option of modulation to capture disease presentations of varied severity [5]. This is a particularly important shortcoming considering that the avascular portion of the femoral head volume in children with LCPD can range from 5 to 100% [21–24], influencing treatment recommendations and patient outcome [2,3,25,26].

Therefore, the objective of this exploratory study was to conduct targeted embolization of vessels supplying the femoral head in juvenile piglets to induce *partial* femoral head ischemia and, by doing so, lay the groundwork for a minimally invasive piglet model of LCPD that can capture the broad range of severity of LCPD seen in pediatric patients. We hypothesized that targeted embolization of specific vessels supplying the porcine femoral head will allow modulation of femoral head ischemia as demonstrated by contrast-enhanced MRI (CE-MRI) and histopathology.

## Materials and methods

### Animals

Eight Yorkshire piglets aged 4–12 weeks weighing 10–22.8 kg were enrolled in this study which was carried out in accordance with the recommendations in the Guide for the Care and Use of Laboratory Animals of the National Institutes of Health. The protocol was approved by the Institutional Animal Care and Use Committee of the University of Minnesota (Protocol Number: 2110-39523A). Using an iterative approach to refine our methods continually, piglets underwent an angiography-guided embolization procedure to obstruct the vascular supply to their femoral heads. Piglets were premedicated with a combination of atropine (0.03 mg/kg), Telazol (tiletamine and zolazepam; 4 mg/kg), and xylazine (2 mg/kg) administered intramuscularly. General anesthesia was induced with intravenous administration of propofol and maintained by inhalation of isoflurane vaporized in oxygen. The vascular anatomy supplying the femoral head was explored using digital subtraction angiography in all piglets with the addition of computed tomography angiography (CTA) with intra-arterial contrast injection in piglets 5–8. Guided by angiographic results, piglets underwent bilateral embolization of the proximal femoral epiphysis. The extent of induced ischemia was assessed immediately post-embolization (piglets 1–4) and/or 7 days following embolization (piglets 1–2 and 4–8) using contrast-enhanced MRI (CE-MRI).

Postoperatively, piglets were treated with flunixin meglumine (1.1 mg/kg, IM) or carprofen (2–3 mg/kg, PO) once or twice daily for three days. Throughout the entire study period piglets were monitored by licensed veterinary technicians twice a day for signs of lameness, surgical site infection and general demeanor. Humane endpoints were used for any of the following reasons: signs of severe, intractable lameness, or surgical site infection that extended beyond 24 hours after initiation of treatment. One piglet died prematurely (piglet 3) due to a perioperative surgical site infection involving the carotid access site, precluding the 7-day MRI. All remaining piglets survived with no to minimal complications (subtle lameness) until the time of planned euthanasia immediately following the 7-day MRI. Immediately after euthanasia (sodium pentobarbital,100mg/kg, IV) femoral heads were harvested, bisected and fixed in 10% neutral buffered formalin for histologic evaluation.

### Embolization procedure

An iterative approach was used to test and refine the embolization procedure targeting blood vessels supplying the femoral head. Embolization was performed on a single animal at a time to assess the reduction in blood flow as well as pathological changes present post-operatively before proceeding with the next animal. Iterative changes made to the embolization approach included the number of vessels targeted, the age of the animal at the time of procedure, and the type of embolic agent used (microspheres [MeritMedical Embospheres 40–120 micron] vs. liquid embolic agents [Lipiodol® or Truefill™]), with or without placement of embolic micro-coils.

**Fig 1:**
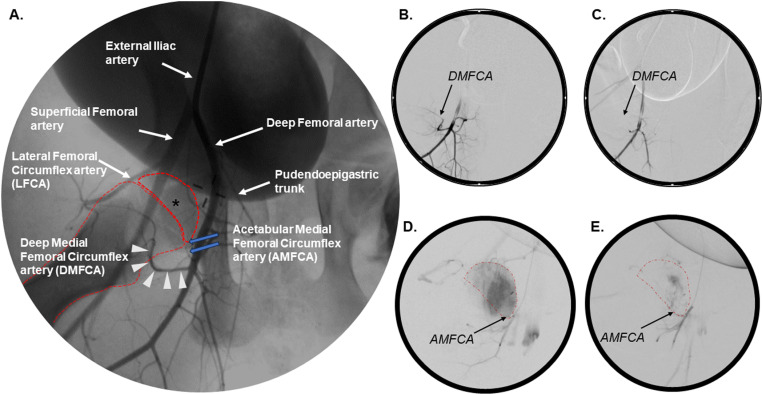
Two-dimensional angiography of the piglet hip pre- and post-embolization. (A) Normal vascular anatomy of the porcine hip, Piglet 1. (B) Sub-selection of the DMFCA demonstrating ascending flow of a contrast agent toward the femoral head, Piglet 4. (C) Absence of perfusion within the DMFCA following its embolization after injection of a contrast agent into the deep femoral artery, Piglet 4. (D) Enhancement of the femoral head after sub-selection of the AMFCA prior to embolization, Piglet 8. (E) Absence of femoral head perfusion after sub-selective injection of a contrast agent into the embolized AMFCA, Piglet 8. Femoral head (*); femoral head and femur (dotted red line); DMFCA (white arrowheads); AMFCA (blue arrows).

In each piglet an open surgical approach was used to introduce a 5 French base angiographic catheter into the carotid artery. Assisted by a guide wire, the external iliac artery was accessed through the descending aorta and an iliac arteriography was performed to identify vessels supplying the femoral head (Fig 1A). Additionally, piglets 5–8 also underwent CTA using 25 mL iodinated contrast media (Visipaque 320, GE Healthcare) to aid further evaluation of the regional blood supply. Individual vessels supplying the femoral head were sub-selected with microcatheters (including use of a balloon microcatheter in piglet 3, to limit off-target embolization) using angiography and occluded with either embolic particles or a liquid embolic agent with or without the placement of embolic coils. Vascular occlusion was deemed successful if there was complete stasis of the contrast agent for a minimum of 5 consecutive heartbeats within the targeted vessel (compare Fig 1B–1C). After completion of the occlusion procedure and closure of the vascular access, piglets were either transported for MRI or allowed to recover.

### MRI

Regions of femoral head ischemia and perfusion were evaluated immediately (within 30 minutes) after the embolization procedure (piglets 1–4) and/or at 7 days post-embolization (piglets 1–2 and 4–8) using subtraction CE-MRI. Immediate post operative evaluation of the embolization using CE-MRI was discontinued after the first four piglets because it was found to be a poor predictor of long-term embolization success. The piglets’ hips were imaged bilaterally in a 3T MRI scanner. T1-weighted TSE images were acquired pre- and post-injection of 0.2 mmol/kg gadoteridol and then subtracted to identify regions of ischemia and perfusion. In piglets 5–8, a higher-resolution 3D GRE subtracted CE-MRI was also acquired.

For each hip, the extent (i.e., percentage volume rounded to the nearest 5%) of femoral head ischemia was determined by an experienced MRI research scientist (CPJ) based on complete absence of signal on subtracted CE-MRI.

### Histology

**Fig 2:**
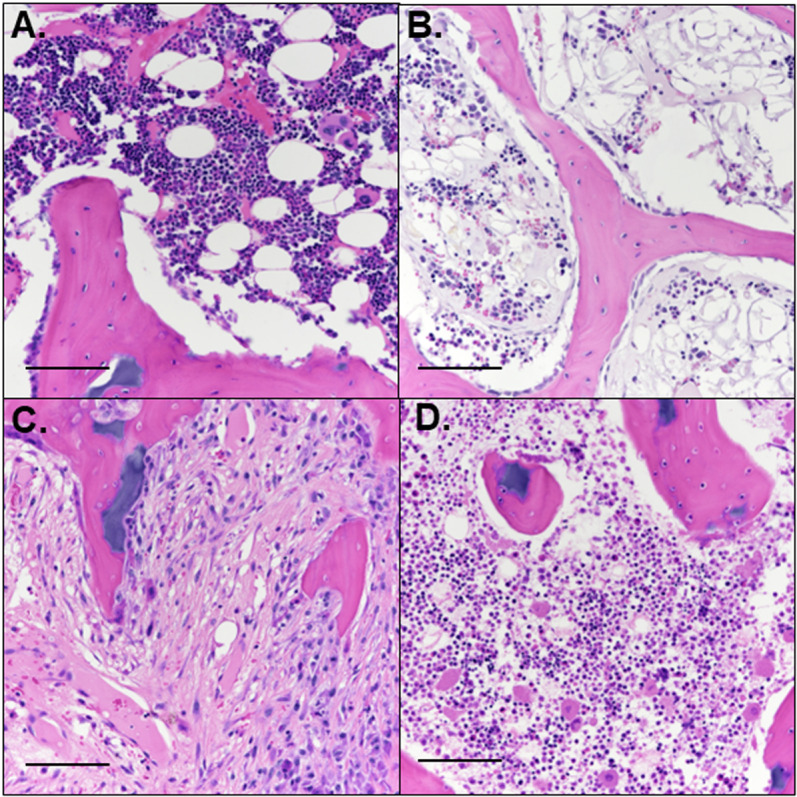
Histologically defined regions of interest. Four distinct patterns were present in the examined sections, including (A) normal marrow and bone, (B) reduced cellularity of marrow without other changes, (C) fibrovascular repair tissue, and (D) necrosis of marrow and/or bone without evidence of repair. H&E, scale bars = 100 µm.

Bisected, formalin-fixed femoral heads were decalcified using 10% ethylenediaminetetraacetic acid, and bread-sliced into 3.0-mm-thick slabs (2 slabs per specimen) in the coronal plane. After routine processing, 5.0-µm-thick sections were obtained from the mid-coronal slab and stained with hematoxylin and eosin (H&E). On average n = 2 histological sections were qualitatively assessed from each slab for the presence of ischemic injury and early repair by a blinded, board-certified veterinary pathologist experienced in musculoskeletal pathology (ARA). Four general patterns were identified: 1 – areas with no evidence of injury or repair (i.e., normal, Fig 2A); 2 – reduced marrow cellularity without other alterations (Fig 2B); 3 – fibrovascular repair with deposition of collagenous matrix and neovascularization (Fig 2C); and 4 – necrosis of bone marrow and/or bone, without any evidence of repair (Fig 2D). The total region of abnormality was calculated as the sum of the areas of complete necrosis, fibrovascular repair, and reduced cellularity for each femoral head. Images were taken at 0.5× and color-coded tracing based on the four defined patterns were applied to each mid-coronal section. Following delineating of regions of interest, the approximate percentage (rounded to the nearest 5%) of the mid-coronal section affected by each pattern was recorded for each sample.

## Results

### Vascular supply to the femoral head

**Fig 3:**
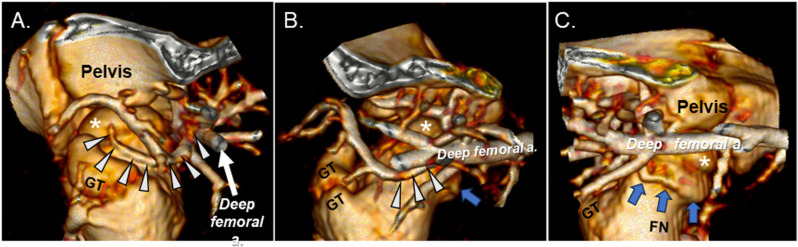
Three-dimensional reconstruction of hip vascular anatomy. (A), (B), and (C) depict the lateral, anterior (cranial), and medial aspects of the hip joint and its vasculature, respectively in a 6-week-old piglet on CT angiography with intra-arterial contrast injection. Femoral head (*); greater trochanter (GT); femoral neck (FN); DMFCA (white arrowheads); AMFCA (blue arrows).

Angiography and CTA identified three arteries, in addition to the *artery of the ligamentum teres*, that were suspected to contribute to the vascular supply of the femoral head in piglets. Embolization of two of these three vessels resulted in ischemia of the proximal femur, confirming their role in supplying the femoral head. The first artery investigated arose from the *deep femoral artery* and travelled in a latero-proximal direction along the intertrochanteric line before entering the femoral epiphysis on its lateral side (Fig 1A and 3A–3B), corresponding to the anatomy of the *deep medial femoral circumflex artery* (DMFCA) in humans and as reported in other preclinical studies [27–29].

**Fig 4:**
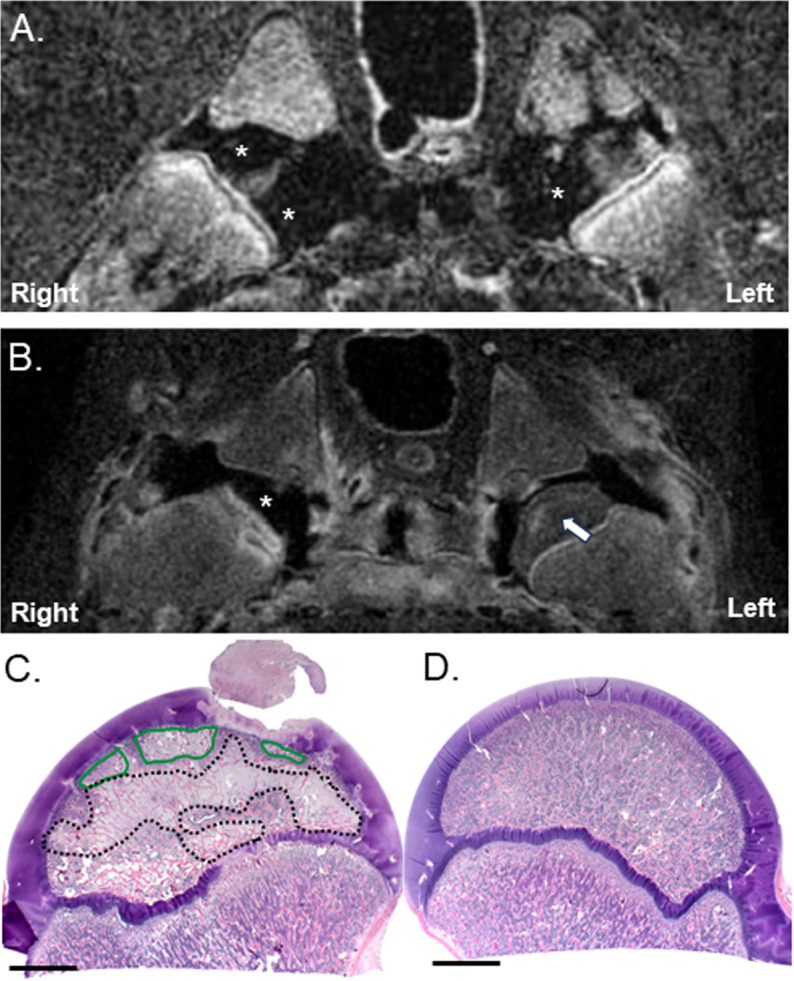
CE-MRI and histology of the hip joints following bilateral embolization of the DMFCA alone in Piglet 1. Subtracted CE-MRI of T1-weighted TSE images acquired either (A) immediately after post-embolization, or (B) 7-days post embolization. Regions of ischemia appear black (*) and regions with perfusion appear either gray or white (arrow). Corresponding histology at 0.5× of the (C) right and (D) left hip obtained after euthanasia at 7-days postoperatively. Areas of fibrovascular repair (black dotted line); areas of necrosis (green line). Scale bars = 5 mm.

In piglets 1–4, embolization of the DMFCA alone resulted in transient, partial ischemia of the secondary ossification center of the femoral head, as demonstrated by CE-MRI conducted immediately post-embolization (Fig 4A and Table 1). At 7 days post-embolization, CE-MRI findings were consistent with partial to nearly complete restoration of femoral head perfusion (Fig 4B and Table 1) with concurrent histologic findings of partial necrosis, fibrovascular repair, and/or reduced cellularity of the femoral head (Fig 4C).

**Table 1:** Embolization procedures and associated femoral head ischemia.

		Left Hip				Right Hip			
					FH Ischemia on CE-MRI (% Volume)			FH Ischemia on CE-MRI (% Volume)	
Pig	Age Mass Sex	Targeted Artery	Embolic agent	Post-Op	7-days	Targeted Artery	Embolic agent	Post-Op	7-days
1	5 wk 13.7 kg Male	DMFCA	6.5 ml of embolic particles	30%	0%	DMFCA	8.5 ml of embolic particles	90%	20%
2	6 wk 12.2 kg Male	DMFCA	6.0 ml of embolic particles	50%	0%	DMFCA	0.5 ml of diluted Lipiodol^®^	10%	0%
3	5 wk 10.0 kg Male	DMFCA	4.5 ml of embolic particles + balloon catheter	80%	N/A	DMFCA	4 ml of diluted Lipiodol^®^	0%	N/A
4	4 wk 10.0 kg M	DMFCA	6 ml of embolic particles	50%	0%	DMFCA	6 ml of embolic particles	50%	20%
5	5 wk 13.8 kg Male	DMFCA	1 ml of diluted Truefill™ + embolic particles	N/A	0%	LFCA + DMFCA	1 ml of diluted Truefill™ + embolic particles	N/A	0%
6	6 wk 11.5 kg Male	DMFCA + AMFCA	20 ml of embolic particles + two coils^1,2^		0%	DMFCA + AMFCA	20 ml of embolic particles + one coil^1^		0%
7	6 wk 14.6 kg Female	DMFCA + AMFCA (failed attempt)	20 ml of embolic particles + coil^3^		0%	DMFCA + AMFCA (failed attempt)	20 ml of embolic particles + coil^4^		0%
8	12 wk 22.8 kg Male	DMFCA + AMFCA	4.5 ml of embolic particles + 1 coil^3^		10%	DMFCA + AMFCA	3 ml of embolic particles + 1 coil^3^		20%

Micro-coil Details: ^1^ Helix ev3 Conscerto coil (2 mm x 4 cm; NV-2–4-HELIX), ^2^ 3D ev3 Concerto coil (2 mm x 2 cm; PV-2–2-3D), ^3^ Helix ev3 Concerto coil (2 mm x 6 cm; NV-2–6-HELIX), ^4^ Helix ev3 Conscerto coil (2 mm x 8 cm; NV-2–8-HELIX). DMFCA = *Deep Medial Femoral Circumflex Artery*; LFCA = *Lateral Femoral Circumflex artery,* AMFCA* = Acetabular Medial Femoral Circumflex Artery*.

**Fig 5:**
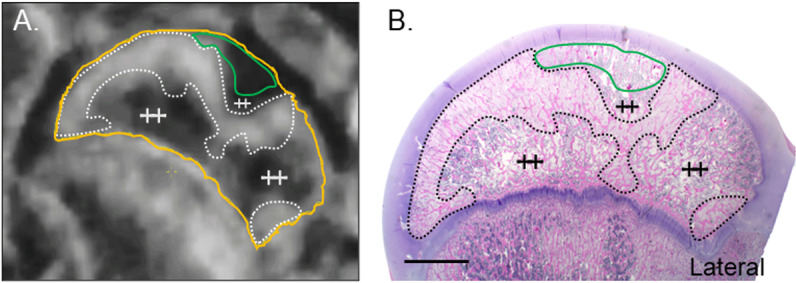
CE-MRI and histology of the right hip following embolization of both the DMFCA and AMFCA in Piglet 8. (A) Subtracted CE-MRI of T1-weighted TSE images acquired 7-days post embolization. (B) Corresponding H&E-stained histology at 0.5×. Secondary center of ossification of the femoral head (yellow line); regions of increased signal on CE-MRI that correspond to regions of fibrovascular repair on histology (dotted line); regions of ischemia on CE-MRI that correspond to acute necrosis without repair on histology (green lines); histologically normal regions of the femoral head (++). Scale bar = 5 mm.

The second artery suspected to provide blood supply to the femoral head arose from the *deep femoral artery* proximal to the DMFCA (Fig 1A). This artery travelled in a cranio-medial direction circumferentially along the femoral neck, most consistent with the *acetabular medial femoral circumflex artery* (AMFCA) described in sheep [29] or the acetabular branch of the medial femoral circumflex artery in humans (Fig 3B–3C) [27]. Subselective angiographic investigation of the AMFCA confirmed the artery’s role in supplying the medial aspect of the femoral head (Fig 1D) and metaphysis. Subsequent embolization of the AMFCA (Fig 1E), in combination with embolization of the DMFCA, resulted in sustained partial ischemia of the femoral head on the 7-day CE-MRI in piglet 8 (Fig 5A).

Selective angiography of the third vessel ostensibly supplying the femoral head, a small artery arising from the *lateral femoral circumflex artery (LFCA)*, resulted in partial enhancement of the proximal femoral epiphysis and metaphysis. However, subsequent embolization of this branch of the *LFCA* in the right hip of piglet 5 had no apparent impact on femoral head perfusion (Tables 1-2), thus it was not investigated further.

The age and size of piglets were found to be critical factors for effective angiography. Visualization of the targeted arteries was challenging in piglets that were young and/or small due to the small lumina of targeted arteries. The transition to a 12-week-old piglet (Piglet 8) enhanced visualization of the DMFCA and AMFCA and facilitated their cannulation and subsequent occlusion.

### Femoral head ischemia and injury

The age, weight, and sex of piglets, the embolic agents used, the vessel(s) targeted, and the percent of femoral head ischemia measured from CE-MRI immediately and 7-days post-embolization are provided in Table 1. Corresponding histologic findings are presented in Table 2.

**Table 2:** Pathologic changes present and regions affected after induction of femoral head ischemia.

Pig	Left Hip					Right Hip				
	% Abnormal	% Necrotic	% Fibrovascular	% Reduced Cellularity	Region Affected	% Abnormal	% Necrotic	% Fibrovascular	% Reduced Cellularity	Region Affected
1	0	0	0	0	None	50	10	40	0	EPI, AECC, LT, PHY
2	35	10	20	5	EPI, AECC	<10	0	0	<10	EPI, MET
3	<25	<10	10	5	EPI, AECC, MET, DIA	<5	0	0	<5	EPI
4	0	0	0	0	None	10	0	0	10	EPI
5	10	0	0	10	EPI	5	0	0	5	EPI, MET
6	50	0	0	50	EPI	30	0	0	30	EPI
7	<5	0	0	<5	EPI	15	0	0	15	EPI
8	30	15	15	0	EPI, AECC, PHY, MET, DIA	60	10	50	0	EPI, AECC, PHY, MET, DIA

Data are represented as the estimated percentage of the femoral head volume using a 0.5× photomicrograph. EPI: epiphysis; AECC: articular-epiphyseal cartilage complex; LT: ligamentum teres; PHY: physis; MET: metaphysis; DIA: diaphysis.

Ischemic necrosis and concurrent presence of fibrovascular repair or decreased cellularity present on histology in all but two of the operated hip joints supported the MRI finding of transient ischemia followed by rapid reperfusion. It was also apparent that the extent of induced ischemia as measured on CE-MRI was higher when embolic particles vs. liquid embolic agents were used with 30–90% vs. 0–10% volume of femoral head involvement, respectively (Table 1). Liquid embolic agents also failed to produce any histological evidence of ischemic necrosis or repair.

The most severe abnormalities were noted after embolization of both the AMFCA and DMFCA in piglet 8 using microspheres in combination with placement of a vascular micro-coil into the DMFCA (Fig 5). Histologic findings were consistent with partial necrosis of both the epiphysis and the adjacent region of the metaphysis. Fibrovascular repair, affecting 50% and 15% of the right and left epiphysis along with portions of the metaphysis, was also present in the histological sections examined.

## Discussion

Our findings explore the feasibility of a minimally invasive piglet model of LCPD that relies on embolization of selected vessels to induce femoral head ischemia. Implementation of angiography and CTA in piglets enabled us to investigate the vascular anatomy and demonstrate that the AMFCA and DMFCA are the primary vessels supplying the femoral head in piglets (Fig 1 and 2). Embolization of the DMFCA alone or in combination with the AMFCA resulted in transient ischemia of a varying portion of the femoral head and accompanying histological changes consistent with those seen in biopsies of children with LCPD [30].

Development of an animal model of partial femoral head ischemia to replicate LCPD as it is seen in most pediatric patients (i.e., avascular femoral head volume 5–100%, [21–24]) requires a thorough understanding of the vascular anatomy supplying the femoral head and an approach that allows selective occlusion of individual vessels. The current study utilized targeted obstruction of the DMFCA alone or in combination with the AMFCA, which are vessels supplying the proximal femoral epiphysis. Previous studies that attempted transarterial embolization techniques to model LCPD provided important information to guide our experiments [31–33]. Injection of embolic particles into the external iliac artery were shown to result in changes limited to the femoral metaphysis [32], and deposition of a gel foam into the femoral artery was only associated with reduction in the numbers of viable osteocytes and marrow cells within the epiphysis and irregularities of the growth plate [33]. More recently, ischemic osteonecrosis and reduced cellularity were noted in 70% of hips 4 weeks after occlusion of the medial femoral circumflex artery (which equates to the deep femoral artery in the current study) using a liquid embolic agent, despite apparent reperfusion of the femoral epiphysis [31].

In the current study, we were able to induce transient femoral head ischemia and histological changes consistent with partial ischemic injury, with embolization of the DMFCA alone or in combination with the AMFCA. Embolization of the DMFCA alone with embolic particles resulted in acute ischemia involving 30–90% volume of the femoral head, followed by rapid restoration of the blood flow by 7 days postoperatively, a process likely occurring through compensatory mechanisms involving the patent AMFCA and the artery of the ligamentum teres. This finding underscores that results of MRI examinations performed immediately post operatively should not be solely relied upon to predict the existence of sustained ischemia, unless they are confirmed by imaging findings obtained ~7 days postoperatively. Interestingly, despite the transient nature of the ischemic event, histological evidence of necrosis and early stages of fibrovascular repair were still observed in all but two of the operated femora, similar to the findings of Cheon et al. [31]. Embolization of both the DMFCA and AMFCA augmented with placement of embolic micro-coils in the oldest pig resulted in sustained ischemia involving 10–20% of the femoral head at 7 days postoperatively. Importantly, the prolonged duration of partial ischemia achieved in this animal was also associated with prominent histologic changes consistent with those described in biopsies obtained from children with LCPD [30], including new bone formation superimposed on dead bone with concurrent presence of regions of reduced cellularity and evidence of fibrovascular repair.

Rapid restoration of femoral head perfusion after extensive deposition of embolic agents were noted in pigs that received repeated MRI examinations one week apart. We attempted to address this complication, with limited success, by gradually escalating the embolization approach. Initially, we administered embolic particles alone, then we experimented with the use of liquid embolics and eventually combined the use of embolic particles and micro-coils. Ultimately, we were able to induce sustained ischemia in four limbs, however it remained limited to ≤ 20% of the femoral head. Regardless, rapid recovery of perfusion is an intriguing finding that raises the important question whether the natural history of LCPD involves a single vascular insult or perhaps entails multiple, consecutive ischemic events. Indeed, evidence from a subset of children with LCPD suggest that ischemia/reperfusion injury associated with multiple ischemic episodes is integral to the pathogenesis of LCPD [20,30]. Future studies utilizing repeated embolization procedures will facilitate understanding how multiple ischemic insults contribute to the pathophysiology of LCPD, enabling a new line of investigation previously inaccessible with the traditional, open surgical model of LCPD.

Piglets aged 4–6 weeks were initially selected for the current study, to mirror the ages of piglets used with the traditional piglet model of LCPD [5,7,8,34]. An additional advantage of 4-6 week old piglets is that their femoral head is comparable in size to that of a four-to-five-year-old child [35]. Unfortunately, in these young piglets, we often found identification and targeted embolization of the AMFCA difficult on conventional angiography due to its small size. Because of this size limitation, eventually we decided to test our approach in an older, 12-week-old pig. Overall, our findings suggest that future iteration of the proposed, minimally invasive model of LCPD should employ piglets aged ~10–12 weeks old, which corresponds to the skeletal age of a 4–6-year-old child, in-line with the clinical presentation of disease (average age 6.5 years) [36]. It is also notable that there is marginal growth of the piglet’s femoral head between 6 weeks and 12 weeks of age. Furthermore, the larger size of these older piglets might also allow accessing more terminal branches of the vascular supply, like the lateral epiphyseal artery that was found frequently affected in clinical patients [20].

The primary limitations of our exploratory study are the iterative approach used to refine the embolization procedure as well as the small sample size and a wide range in the age and size of piglets investigated, which precluded demonstration of repeatability of the model and quantitative comparisons. Despite these limitations, we were consistently able to identify and embolize the targeted vessels in all but one piglet (piglet 7) and make important observations to inform further refinement of the model, including: 1) embolizing both the AMFCA and DMFCA resulted in a greater degree of pathologic changes at 7 days post-operatively than embolization of the DMFCA alone; 2) combining embolic particles with microcoils is advantageous compared to the use of liquid embolics; and 3) using an older piglet (12 weeks of age) improves the access to the targeted vessels. Future studies aiming to establish a minimally invasive model of LCPD must therefore also investigate the role developmental age (and associated compositional changes in the femoral head) plays in the development and progression of LCPD after ischemic injury. An additional limitation of our study is that clinical staging and classifications of LCPD such as the modified Waldenström staging [37] and the Lateral Pillar classification [38] were not utilized. Radiographic changes consistent with LCPD become apparent at 3-week post-ischemia in the traditional model [7], and thus were not anticipated in our 7-day study period for this proof-of-concept study. Future studies which include longer follow-up durations are warranted to allow evaluation of the minimally invasive piglet model of LCPD using clinically relevant staging and classification methods. Lastly, it must be acknowledged that although the animal’s discomfort associated with the bilateral embolization procedure was well controlled with administration of non-steroidal anti-inflammatory drugs for a 72-hour period, one of the piglets died prematurely due to a peracute surgical site infection. Postmortem evaluation confirmed that this complication was due to a surgical error that resulted in iatrogenic injury to the esophagus during closure of a right sided paratracheal incision used to access the right carotid artery. This complication was averted in all subsequent cases by performing the embolization procedure using the left carotid artery, away from the esophagus located on the right side of the neck.

In conclusion, our study demonstrates the feasibility of a minimally invasive piglet model of femoral head ischemia in which the severity of induced lesions ranges from <5–60% femoral head involvement. Considering that the extent of avascularity ranges from 5–100% in children with LCPD [21–24], an animal model with the potential of capturing mild to moderate disease severity is necessary to complement the traditional model that is best suited to replicate the most severe cases of LCPD.
